# P-1357. Molecular Mechanisms of Carbapenemase Genes Dissemination in *Klebsiella pneumoniae* in Two Colombian Cities (2018-2021)

**DOI:** 10.1093/ofid/ofae631.1534

**Published:** 2025-01-29

**Authors:** Nalleth Danieyis Bolano-Ardila, Elsa De La Cadena, José Y Rodríguez, Adriana Marin, Maria Virginia Villegas, Maria F Mojica

**Affiliations:** Universidad Popular del Cesar, Valledupar, Cesar, Colombia; Universidad El Bosque, Bogota, Distrito Capital de Bogota, Colombia; Centro de Investigaciones Microbiológicas del Cesar, Valledupar, Cesar, Colombia; Clínica General del Norte, Barranquilla, Atlantico, Colombia; Universidad El Bosque, Bogota, Distrito Capital de Bogota, Colombia; Case Western Reserve University, Cleveland, Ohio

## Abstract

**Background:**

Colombia has been endemic for KPC for decades, mainly due to the clonal dissemination of *bla*_KPC-3_ among carbapenemase-producing *K. pneumoniae* (CP-Kp) sequence type (ST) 258 and plasmid dissemination of *bla*_KPC-2_ among different species of Gram-negatives. However, a recent surge in the detection of *bla*_NDM_ among CP-Kp has raised concerns. Herein, we aimed to understand the dynamics of transmission of carbapenemase genes among CP-Kp clinical isolates in two cities of Colombia.

**Fig. 1.**
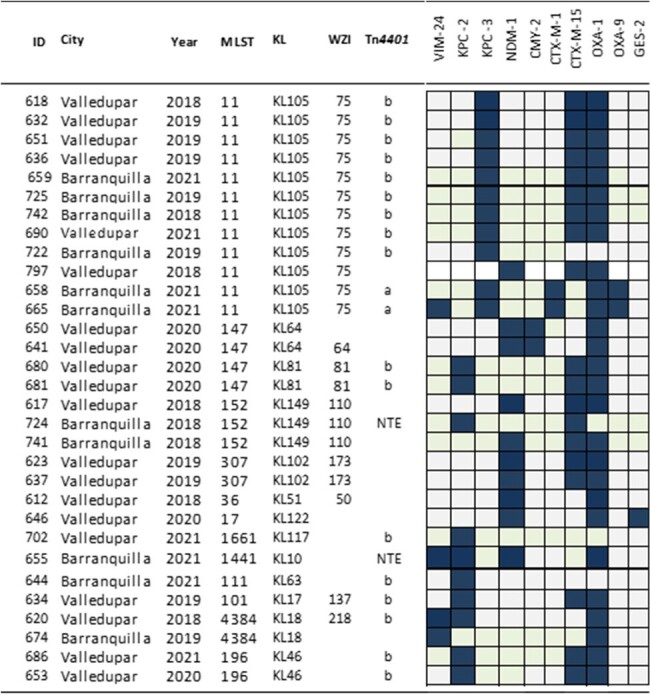
Molecular characterization of 31 CP-Kp collected between 2018 - 2019. MLST: Multi-locus sequence type; KL: capsule locus type; wzi: wzi alleles. Blue squares represent presence, gray absence.

**Methods:**

We conducted a surveillance study in 7 hospitals in two Colombian cities, Valledupar and Barranquilla. Isolates were collected between 2018 and 2021, identified, and typed initially using rep-PCR. A subset of isolates was chosen for Whole Genome Sequencing on the Illumina platform based on pulsed-field gel electrophoresis (PFGE) pulsotypes. De-novo assembly and maximum likelihood phylogenetic analyses were performed.

**Fig.2.**
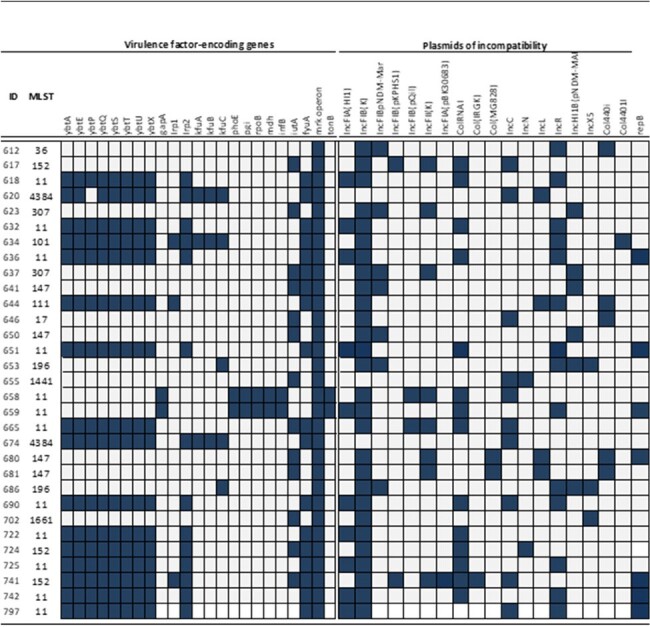
Virulence determinants and incompatibility groups of plasmids found in 31 CP-Kp. Blue squares represent presence, gray absence.

**Results:**

A total of 89 CP-Kp isolates were recovered from infected patients, the majority of which produced KPC (64/89; 72%) and NDM (20/89; 22.5%); co-production of KPC and NDM or VIM was detected in 4/89 (4.5%) isolates. Following PFGE, 31 isolates were sequenced. **Fig. 1** shows the relationship between the 12-sequence type (STs) identified and specific *bla* genes. Notably, ST11 was associated with *bla*_KPC-3_, whereas *bla*_KPC-2_ and *bla*_NDM-1_ were associated with several STs. One isolate harbored 3 carbapenemase genes, *bla*_KPC-2_, *bla*_NDM-1_, and *bla*_VIM-24_. Among other *bla* genes, *bla*_OXA-1_ and *bla*_CTX-M-15_ were found in 81% and 61% of the isolates, respectively. Most (81%) of *bla*_KPC-2_ and *bla*_KPC-3_ genes were found within a Tn*4401*b structure, followed by Tn*4401*a and non-Tn44 elements (NTE_KPC_; 9.5% each). Most isolates (90%) carried a complete *mrk* operon, associated with biofilm production, while the genes encoding siderophores *fyuA* and *ybt* were present in 58% and 51% of the isolates, respectively. Lastly, IncF plasmids were the most common type found among CP-Kp (**Fig.2**)

**Conclusion:**

Although KPC remains the most common carbapenemase among CP-Kp, the rise of NDM associated with plasmid dissemination within different STs is concerning. Moreover, detecting CP-Kp isolates co-producing carbapenemases of different molecular classes poses a clinical challenge, as therapeutic options are scarce.

**Disclosures:**

**Maria Virginia Villegas, MD**, bioMérieux: Advisor/Consultant|bioMérieux: Grant/Research Support|Merck Sharp and Dohme: Advisor/Consultant|Merck Sharp and Dohme: Grant/Research Support|Pfizer: Advisor/Consultant|Pfizer: Grant/Research Support|WEST: Advisor/Consultant|WEST: Grant/Research Support

